# Theta-band phase locking during encoding leads to coordinated entorhinal-hippocampal replay

**DOI:** 10.1016/j.cub.2023.09.011

**Published:** 2023-11-06

**Authors:** Diogo Santos-Pata, Caswell Barry, H. Freyja Ólafsdóttir

**Affiliations:** 1 Division of Natural and Applied Sciences, Duke Kunshan University, Duke Institute for Brain Sciences, Kunshan 215316, Jiangsu, China; 2 Research Department of Cell and Developmental Biology, University College London, London WC1E 7JE, UK; 3 Donders Institute for Brain, Cognition & Behaviour, Radboud Universiteit, 6525 XZ Nijmegen, the Netherlands

**Keywords:** hippocampus, phase locking, place cells, medial entorhinal cortex, replay, sharp-wave ripples, theta oscillations, learning

## Abstract

Precisely timed interactions between hippocampal and cortical neurons during replay epochs are thought to support learning. Indeed, research has shown that replay is associated with heightened hippocampal-cortical synchrony. Yet many caveats remain in our understanding. Namely, it remains unclear *how* this offline synchrony comes about, whether it is specific to particular behavioral states, and how—if at all—it relates to learning. In this study, we sought to address these questions by analyzing coordination between CA1 cells and neurons of the deep layers of the medial entorhinal cortex (dMEC) while rats learned a novel spatial task. During movement, we found a subset of dMEC cells that were particularly locked to hippocampal LFP theta-band oscillations and that were preferentially coordinated with hippocampal replay during offline periods. Further, dMEC synchrony with CA1 replay peaked ∼10 ms after replay initiation in CA1, suggesting that the distributed replay reflects extra-hippocampal information propagation and is specific to “offline” periods. Finally, theta-modulated dMEC cells showed a striking experience-dependent increase in synchronization with hippocampal replay trajectories, mirroring the animals’ acquisition of the novel task and coupling to the hippocampal local field. Together, these findings provide strong support for the hypothesis that synergistic hippocampal-cortical replay supports learning and highlights phase locking to hippocampal theta oscillations as a potential mechanism by which such cross-structural synchrony comes about.

## Highlights

• Spatially tuned dMEC cells display mixed coupling to hippocampal theta oscillations
• Theta-coupled dMEC cells are preferentially coordinated with hippocampal replay
• dMEC-hippocampal replay coordination is specific to rest periods
• The coordination increases in tandem with the animals’ ability to carry out a task

## Teaser

Santos-Pata et al. report that spatially modulated dMEC cells that are locked to hippocampal theta-band oscillations preferentially coordinate with hippocampal replay recorded during rest periods. The replay coordination only emerges after an animal’s first encounter with the task environment and then shows a learned-related increase.

## Introduction

The establishment of long-term memories is thought to be underpinned by precisely timed interactions between hippocampal and cortical circuits during periods when hippocampal cell sequences, reflecting wakeful experiences, are reactivated (“replayed”).^1^^,^^2^^,^^3^^,^^4^ Specifically, coordinated hippocampal-cortical replay is proposed to gradually establish cortical memory traces and, thereby, the creation of robust, persistent memories. Indeed, replay periods are associated with heightened hippocampal-cortical communication,^5^^,^^6^^,^^7^^,^^8^ and cortical cells have been found to replay synergistically with hippocampal cells.^5^^,^^9^^,^^10^

In previous work we showed that during hippocampal replay recorded during rest, CA1 place cells and grid cells of the deep layers of the medial entorhinal cortex (dMEC)—the principal cortical output region of the hippocampus—are functionally coordinated, depicting similar spatial positions.^5^ Yet the processes during encoding that lead to this coordinated hippocampal-dMEC replay—possibly paving the way for future long-term memory formation—remain largely unknown. Further, whether this cross-structural synchrony reflects learning or simply is the result of pre-existing biases in hippocampal-cortical circuits has hitherto not be investigated. Finally, recent years have seen a proliferation of studies that indicate replay may serve numerous functions in cognition—including learning,^8^^,^^11^^,^^12^ planning,^13^^,^^14^ and memory retrieval.^15^^,^^16^ Importantly, the function replay serves is potentially segregated by behavioral state, with replay occurring during rest/sleep (offline periods) primarily thought to support learning.^2^^,^^14^^,^^17^ Yet whether the dMEC participates and synchronizes with all types of replay or whether this coordination is specific to offline periods is unknown. In this study, we sought to address these questions.

We found that dMEC cells with activity that was strongly modulated at theta-band frequencies were preferentially recruited during, and functionally coordinated with, hippocampal replay trajectories of a familiar track. Conversely, dMEC cells that were not strongly theta-modulated were no more coordinated with CA1 replays than expected by chance. The coordination between theta-modulated dMEC and CA1 was only found during offline replay events emitted while animals rested, not during simple pauses in online activity, and peaked 10 ms after CA1 replay—suggestive of a role in memory consolidation. Importantly, the preferential coordination of theta-modulated dMEC cells with CA1 replay could not be explained by functional or activity differences between the two dMEC sub-groups. Finally, dMEC-hippocampal replay coordination of the task environment showed a robust learning-related effect, emerging only after an animal’s first exposure to the track and then strengthening in tandem with that animal’s capability to carry out the task fluently.

Taken together, these findings suggest that oscillatory coupling, particularly in the theta-band, between hippocampal and entorhinal cells during encoding periods, may be responsible for their offline synchronization. Further, the time lag of the dMEC-hippocampal replay synchrony, the accentuated coordination observed during offline periods, and the relationship between dMEC-hippocampal replay synchrony and task acquisition together provide compelling evidence for the hypothesis that this cross-structural synchronization represents a hallmark of learning. Finally, as coupling of CA1 cells to hippocampal theta oscillations is implicated in the generation of hippocampal theta sequences,^18^^,^^19^^,^^20^^,^^21^^,^^22^^,^^23^^,^^24^ we speculate that the dMEC theta phase locking may reflect the emergence of distributed hippocampal-cortical theta sequences and that this distributed temporal code may facilitate the formation of long-term memories.

## Results

**Figure 1:**
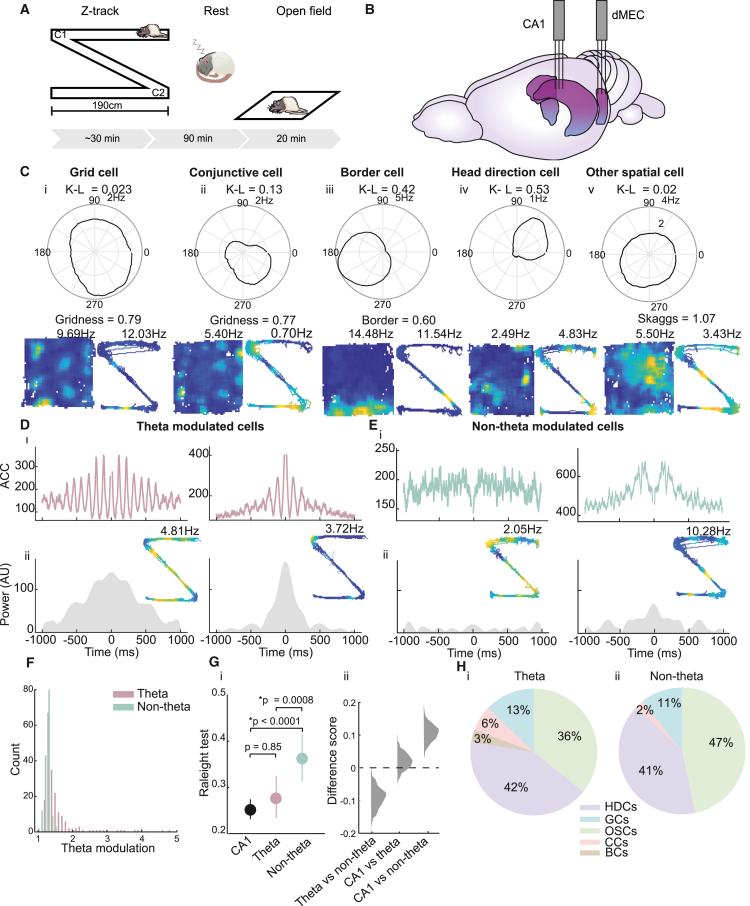
Spatial and oscillatory characterization of dMEC cells (A) Schematic overview of the study. Rats shuttled back and forth between the ends of a Z-shaped track (left), then rested for 90 min in a separate environment (middle), and then completed a 20-min foraging session in the open field. (B) Two tetrode arrays were implanted in the CA1 and dMEC. (C) Functional cell types recorded in dMEC. Top: polar plot showing directional tuning (directional tuning is indicated above each polar plot [K-L]). Bottom: ratemaps showing spatial modulation in the open field and on the Z-track; title shows grid score (20) (far left, middle left), border score (21) (middle), or Skaggs information (22) (bits/spike, far right), and the peak rate (Hz) is shown on the right, above each ratemap. Spatial cell types from left to right: grid cell, conjunctive grid × head direction cell, border cell, head direction cell, other spatial cells. (D) (i) Auto-correlograms for two dMEC cells that show theta-band activity modulation. (ii) Power in the theta band (5–12 Hz) for the autocorrelogram in (i). Inset: ratemap for cell on the Z-track. (E) Same as (D), but for dMEC cells that do not show significant theta-band activity modulation. (F) Histogram distribution of theta modulation scores for the modulated and non-modulated sub-groups. (G) (i) Mean phase locking to hippocampal theta for CA1 (black), dMEC theta-modulated (pink), and non-modulated (green) cells. Error bars show 95% confidence interval (CI). (ii) Histogram distribution of bootstrapped difference scores. (H) Pie chart showing contribution of different spatial cell types to the theta-modulated and non-theta-modulated dMEC sub-groups. HDCs, head direction cells; GCs, grid cells; OSCs, other spatial cells; CCs, conjunctive cells; BCs, border cells. See also Figures S1 and S2.

We analyzed co-recorded CA1 place cells (mean = 34 [SD = 17.7], 14–71 per session) and excitatory cells from the deep layers (V/VI) of the MEC (dMEC) (mean = 14.41 [SD = 4.67], 7–23 per session) across multiple days (Figure 1B) in six rats, while the animals ran on a Z-shaped track for food reward (RUN), rested in a cylinder-shaped environment outside the maze environment (REST), and finally explored an open-field environment (Figure 1A; as described previously in Ólafsdóttir et al.^5^). Recordings took place over 2–6 days. As the identification of replay trajectories depends on cells displaying spatially confined activity, we limited our analyses to dMEC cells that fell into one of the following functional classes: grid cells,^25^ head direction cells,^26^ conjunctive grid and head direction cells,^27^ border cells,^28^^,^^29^ and other spatial cells (Figure 1C; see STAR Methods for cell classification).

### Theta modulation in dMEC neurons

To identify theta-modulated dMEC cells, we constructed an auto-correlogram for each dMEC cell spike train and assessed the power in the autocorrelogram in the theta band (5–12 Hz) relative to a broadband (20–125 Hz). Cells whose theta-to-broadband ratio exceeded the 97.5^th^ percentile of its own shuffle distribution (based on random permutation of spike times) were considered theta-modulated cells (STAR Methods). Just over half of the dMEC cell population qualified as being theta-modulated on this measure (56.18%, 191 cells; see Figures 1D–1F and S1 for examples). Similarly, theta-modulated dMEC cells showed significantly stronger phase locking to hippocampal theta compared with non-theta-modulated cells (Raleigh’s test of uniformity [lower value means higher locking]: theta-modulated cells = 0.28 [SD = 0.31], non-theta-modulated cells = 0.36 [SD = 0.31], p = 0.008, based on computing difference scores between bootstrapped phase locking scores for the two sub-groups; Figure 1G). Indeed, the phase locking to hippocampal theta exhibited by these dMEC cells was not different to that of CA1 place cells (Raleigh’s test of uniformity = 0.25 [SD = 0.30], p = 0.85; Figure 1G), suggesting that the oscillatory coupling observed in this dMEC sub-group may be synchronized with the hippocampal LFP.

Although both dMEC groups contained a mixture of different spatial cell types (see Figure 1H for a breakdown of the different spatial cell types for the two dMEC groups), we found some differences in the representation of individual functional cell categories between the two groups. Specifically, we found that grid cells, conjunctive grid cells, and border cells made up a (marginally) greater proportion of theta-modulated cells compared with non-theta-modulated cells (grid cells, theta-modulated = 19.25%, non-theta-modulated = 12.84%, p = 0.056; conjunctive grid cells, theta-modulated = 6.42%, non-theta-modulated = 2.03%, p = 0.019; border cells, theta-modulated = 3.21%, non-theta-modulated = 0%, p < 0.0001, bootstrapped proportions; Figures S2A, S2D, and S2E). Conversely, other spatial cells contributed to a greater extent to the non-theta-modulated sub-group (35.83% of theta-modulated and 46.62% of non-theta-modulated cells, p = 0.023; Figure S2C). The proportion of head direction cells did not, however, differ between the two groups (41.71% of theta-modulated and 40.54% of non-theta-modulated cells; Figure S2B). These differences are controlled for in subsequent analyses.

### Theta-modulated dMEC cells show enhanced coordination with hippocampal replay

Next, we sought to investigate whether coupling to theta-band oscillations influences dMEC cells’ coupling to hippocampal replay events. To this end, we first analyzed the participation of the two dMEC groups—theta-modulated and non-theta-modulated—in candidate replay events. As this analysis does not require replay events to express coherent spatial trajectories, all hippocampal MUA events submitted for replay trajectory analysis were included (“candidate” replay events; STAR Methods). Further, to ensure that the difference in baseline activity would not influence the results, we computed normalized replay participation scores. Namely, we permuted the timing of candidate replay events—by adding a random time shift to individual events—and determined the proportion of events in which each cell was active in these shuffled events. This process was repeated 100 times and the average shuffle score then computed. The proportion of events a cell was active in the real data was then divided by the mean of the shuffled data (a value above 1 indicates the cell is more active during events than expected by chance). To note, we did not observe any difference in average activity of the two dMEC cell groups on the track (Figure S4A; theta, 1.25 Hz [SD = 0.93]; non-theta, 1.13 Hz [SD = 1.01]; p = 0.13) or during rest periods (Figure S4C; theta, 0.92 Hz [SD = 0.90]; non-theta, 0.88 Hz [SD = 0.89]; p = 0.36), nor did we find that activity on the track correlated with replay participation scores (Figure S4B; r = −0.097, p = 0.089).

**Figure 2:**
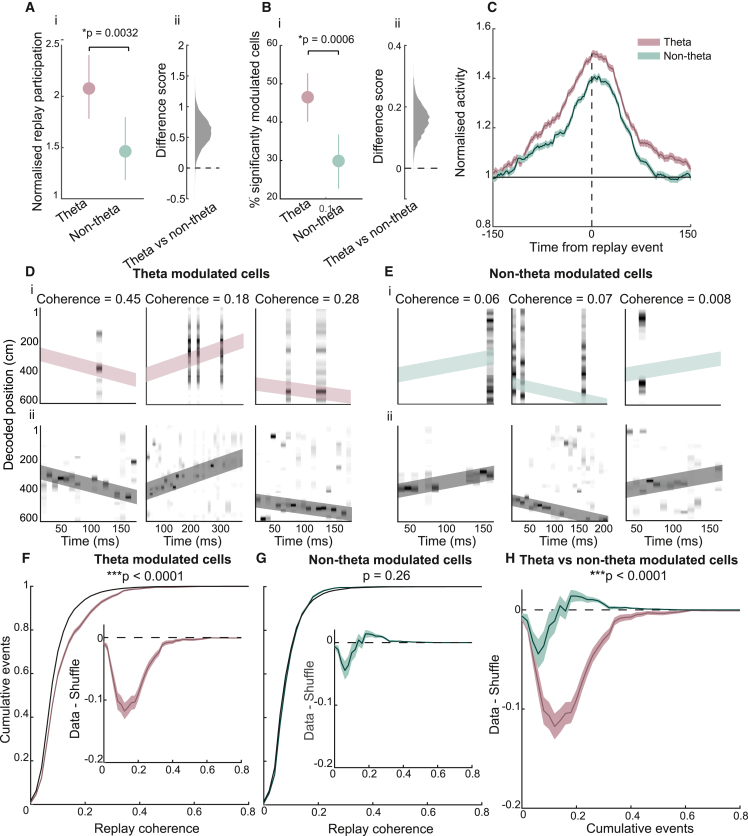
Theta-modulated dMEC cells preferentially coordinate with hippocampal replay (A) (i) Mean-normalized replay participation for dMEC theta-modulated and unmodulated cells. Note, a value above 1 indicates cells are more active during candidate replay than expected by chance. Error bars show 95% CIs. (ii) Histogram distribution of bootstrapped difference scores. (B) (i) Mean proportion of dMEC cells that are significantly modulated by candidate replay events. Error bars show 95% CI. (ii) Histogram distribution of bootstrapped difference scores. (C) Peri-stimulus-time histogram centered on the middle of candidate CA1 replay events for theta-modulated and non-theta-modulated dMEC cells. Shaded area shows SEM. (D) Representative replay trajectories with dMEC activity. (i) Bottom: position reconstruction based on CA1 spikes with best-fit line superimposed (dark gray diagonal line). Top: position reconstruction based on spikes from dMEC theta-modulated cells, CA1 best-fit line is fitted onto dMEC decoding (pink diagonal line). Title shows dMEC-CA1 replay coherence, indicating the proportion of variance in the dMEC decoding that can be accounted for by the CA1 replay trajectory (pink diagonal). (E) Same as (D) but for non-modulated dMEC cells. (F) Cumulative distribution of replay coherence scores for dMEC theta-modulated cells, shaded area shows one SD. Black line shows cumulative distribution of a cell ID shuffle. Inset: difference between the data and shuffle distribution. (G) Same as (F) but for non-modulated dMEC cells. (H) Data-shuffle distributions for the two dMEC cell types. See also Figure S3.

We observed that both dMEC sub-groups were significantly more likely to participate in candidate replay events than expected by chance (theta-modulated cells = 2.10 [SD = 2.14], p < 0.0001; non-theta-modulated cells = 1.46 [SD = 1.83], p = 0.0001, based on computing the difference between bootstrapped participation scores; STAR Methods; Figure 2A). However, theta-modulated cells showed significantly stronger modulation by candidate events compared with their non-theta-modulated counterparts (p = 0.004; Figure 2A). This effect was replicated with a mixed-effects model where animal ID was included as a random effect (*t*_(314)_ = 2.54, p = 0.01). Further, a significantly greater proportion of theta-modulated cells showed replay participation that significantly exceeded chance levels relative to their own shuffle distribution compared with non-theta-modulated cells (theta-modulated cells = 46.50% [SD = 3.21], non-theta-modulated cells = 30.18% [SD = 3.60], p = 0.0006; Figure 2B), a result that was also replicated with a mixed-effects model (*t*_(315)_ = 5.58, p < 0.0001). Similarly, replay modulation, as measured by normalized replay activity rates (i.e., average number of spikes emitted during candidate events normalized by chance), revealed the same pattern of results (mean normalized activity: theta-modulated cells = 2.17 [SD = 2.44] p < 0.0001, non-theta-modulated cells = 1.55 [SD = 2.07], p = 0.0002; theta versus non-theta-modulated, p = 0.0096; percentage of significantly modulated cells: theta-modulated = 44.85% [SD = 3.18], non-theta-modulated = 9.54% [SD = 3.50]; theta-modulated versus non-theta-modulated, p = 0.001; Figures S4A and S4B). Finally, a peri-stimulus time histogram (PSTH) analysis showed that both cell groups displayed a similar temporal profile in relation to hippocampal replay events, their activity peaking ∼10 ms after CA1 (Figure 2C). Thus, both dMEC cells groups showed a lagged activity increase during hippocampal replay, but the degree of modulation was significantly stronger for the dMEC cells displaying theta-band activity rhythmicity.

Next, we investigated whether theta-modulated dMEC cells showed stronger functional coordination with hippocampal replay than their non-theta-modulated counterparts (Figures 2D and S2E; see STAR Methods and Ólafsdóttir et al.^5^ for explanation of methods). To this end, we assessed whether the hippocampal replay trajectory accounted for more variance in the dMEC decoding than expected by chance (i.e., if darker shades of gray in the dMEC decoding centered around the pink and green lines; Figures 2C and 2D, respectively). This measures the similarity of the spatial representation of hippocampal and dMEC cells during replay, with above-chance similarity suggesting functional coordination between dMEC and hippocampal cells.

We observed a striking coordination between CA1 and dMEC theta-modulated cells (exceeding the 97.5^th^ percentile of a shuffle distribution obtained by permuting cell IDs, bootstrapped area under the curve [AUC] of data shuffle: p < 0.0001; STAR Methods; Figure 2F). Conversely, non-theta-modulated dMEC cells were not coordinated with CA1 cells during replay (Figure 2G; data versus shuffle, p = 0.26) and were less coordinated than the theta-modulated cells (based on computing the difference between bootstrapped replay coordination scores of theta-modulated and non-theta-modulated cells; Figure 2H; p < 0.0001). Importantly, these results were replicated with a mixed-effects model that controlled for animal-specific effects (*t*_(2,342)_ = 7.93, p < 0.0001). Similar results were obtained when other shuffle regimes routinely employed in the field,^5^^,^^30^^,^^31^ such as a spatial firing field shuffle (theta-modulated versus non-theta-modulated replay coordination: p < 0.0001; Figure S3D) or a spike-time shuffle (theta-modulated versus non-theta-modulated replay coordination: p < 0001; Figure S3E) were used. To note, the results were replicated when a more stringent threshold for dMEC theta modulation was used (99^th^ percentile, theta-modulated versus non-theta-modulated replay coordination, p < 0.0001; Figure S3C), when the analysis was limited to events that contained at least five dMEC spikes^30^ (p < 0.0001; Figure S4F), when the number of theta-modulated and non-theta-modulated cells was matched (p < 0.0001; STAR Methods), and when the analysis was done separately for forward and reverse replay events (forward replay, p < 0.0001; reverse replay, p < 0.0001; Figures S3G and S3H). Furthermore, the results were replicated for each of the three animals that individually had >10 cells in each of the two dMEC sub-groups (R2335, p < 0.0001; R2336, p = 0.0008; R2337, p < 0.0001; Figures S3I–S3K).

**Figure 3:**
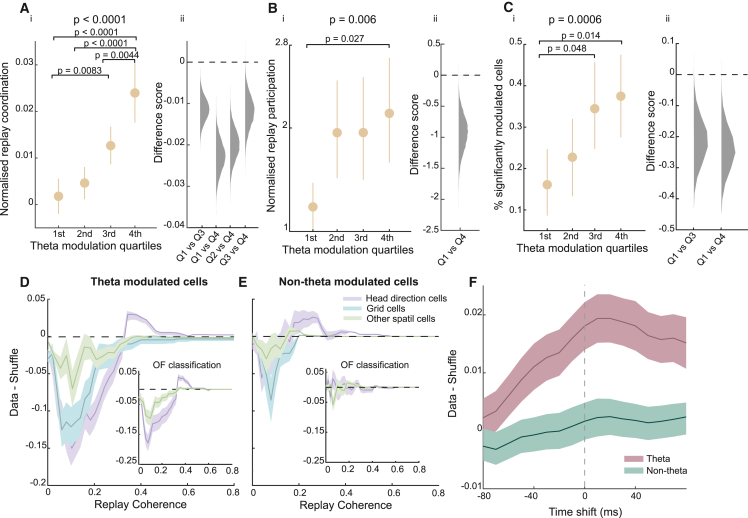
Cell-type and temporal specificity of dMEC-hippocampal replay coordination (A) (i) Mean normalized (data-shuffle) replay coordination for dMEC cells belonging to different theta modulation quartiles. Error bars show 95% CI. (ii) Frequency distribution for bootstrapped difference scores for pairwise comparisons that exceeded the threshold for statistical significance after a Bonferroni correction. (B and C) (i and ii) Same as (A) but for replay participation scores (B) and proportion of dMEC cells significantly modulated by hippocampal replay (C). (D) Normalized (data-shuffle) coordination between theta-modulated dMEC and hippocampal cells during replay trajectories for the different spatial cell types. Error bars show 1 SD. Inset: replay coordination for head direction and other spatial cells when cell classification was done using activity on the track. (E) Same as (D) but for non-theta-modulated dMEC cells. (F) Mean dMEC-hippocampal replay coordination for different time shifts of dMEC spikes. See also Figure S4.

Moreover, we observed dMEC-hippocampal replay coordination and participation of dMEC cells in candidate replay events increased linearly with the degree of theta modulation exhibited by dMEC cells. Specifically, we divided the dMEC cells into quartiles based on their theta modulation score, and for each quartile, we computed the coordination and participation with hippocampal replay. Using a linear mixed-effects model, where animals were included as random effects, we observed a significant effect of quartile (replay participation, *t*_(314)_ = 2.75, p = 0.006; % significantly modulated, *t*_(315)_ = 3.47, p = 0.00059; replay coordination, *t*_(2,798)_ = 6.3, p < 0.0001; Figures 3A–3C), and we found that cells belonging to lower quartiles displayed lower coordination with replay compared with cells belonging to higher quartiles (replay participation, 1^st^ versus 4^th^ quartile, p = 0.027; % significantly modulated, 1^st^ versus 3^rd^ quartile, p = 0.048; 1^st^ versus 4^th^ quartile, p = 0.014; replay coordination, 1^st^ versus 3^rd^ quartile, p = 0.0083; 1^st^ versus 4^th^ quartile, p < 0.0001; 2^nd^ versus 4^th^ quartile, p < 0.0001; 3^rd^ versus 4^th^ quartile, p = 0.0044; p values Bonferroni corrected; Figures 3A–3C).

### Replay coordination is not determined by activity or functional differences between theta-modulated and non-theta-modulated dMEC cells

Here, we sought to investigate whether the differences in replay coordination for the theta-modulated and non-theta-modulated sub-groups could be explained by activity and/or functional differences between the two dMEC sub-groups. First, we assessed whether the groups differed in overall activity and the amount of spatial information carried by individual spikes on the track (Skaggs information). The two groups displayed similar mean activity rates during rest periods (theta, 0.92 Hz [SD = 0.90]; non-theta, 0.88 Hz [SD = 0.88]; p = 0.36; Figure S4C) and on the track (theta, 1.25 Hz [SD = 0.93]; non-theta, 1.13 Hz [SD = 1.01]; p = 0.13; Figure S4A), and spatial information also did not differ (theta-modulated cells, 1.65 [SD = 0.74]; non-theta-modulated cells, 1.57 [SD = 0.61]; p = 0.85; Figure S4E). Next, we sought to investigate whether the theta-modulated sub-group was more likely to have overlapping spatial firing fields on the Z-track with co-recorded CA1 cells than the non-theta-modulated group. To this end, we computed the average spatial correlation between dMEC cells and all co-recorded CA1 cells. On average, both cell groups showed a low correlation with CA1 cells (theta-modulated, r = 0.00033; non-theta-modulated, r = −0.0077), which did not significantly differ from each other (p = 0.084, bootstrapped means; Figure S4D).

Further, the size of the spatial firing field of the dMEC cells could potentially influence replay coordination. Thus, we computed the average field size of the two dMEC sub-groups. The spatial firing field of theta-modulated cells was on average 142.17 cm (SD = 149.69) long, whereas that of non-theta-modulated cells was 171.23 cm (SD = 156.04) long, a difference we found to be statistically significant: p = 0.043 (Figure S4F). Note, this analysis includes all the different functional cell types (see Figure S4G for field sizes of individual dMEC functional cell types). To ensure this difference could not account for the preferential replay coordination of theta-modulated dMEC cells, we repeated the replay coordination analysis using a maximum field size criterion that equalized the average field size between the groups. To note, we used two different field size thresholds (150 and 100 cm), both of which lead to average field sizes that did not differ between the two groups (150 cm control, theta-modulated cells = 68.98 cm [SD = 31.22], non-theta-modulated cells = 74.87 cm [SD = 31.41], p = 0.18; 100 cm control, theta-modulated cells = 57.64 cm [SD = 20.14], non-theta-modulated cells = 61.28 cm [SD = 21.21], p = 0.26). Importantly, controlling for field size did not affect the preferential replay coordination we observed for the theta-modulated sub-group (theta versus non-theta-modulated coordination, p < 0.0001 for both field thresholds; Figures S4H and S4I).

As mentioned above, some differences exist in the type of spatial cells that make up the theta-modulated and non-theta-modulated sub-groups. This could impact our results if, for example, one spatial cell category is more likely to be coordinated with hippocampal replay than another. To control for this potential confound, we carried out the replay coordination analysis separately for the different spatial cell groups. To note, as we recorded from only 15 conjunctive head direction cells (4.48%) and six border cells (1.79%), this analysis focused on grid (n = 55, 16.42%, including conjunctive cells), head direction (n = 138, 41.19%), and other spatial cells (n = 136, 40.60%). In all cases, we found significantly stronger coordination with hippocampal replay for the theta-modulated subset of each group compared with the non-theta-modulated subset (based on computing the difference between bootstrapped theta-modulated and non-theta-modulated replay coordination scores: grid cells, p < 0.0001; head direction cells, p < 0.0001; other spatial cells, p = 0.046; Figures 3D and 3E). The same results were obtained when cells were classified as head direction or other spatial cells, using activity on the track rather than activity in the open field (Figures 3D and 3E, inset). Thus, the near-exclusive coordination of theta-modulated dMEC cells with CA1 replay trajectories cannot be explained by some intrinsic differences between the two cell groups. Rather, we propose that their replay synchronization results from their shared oscillatory coupling with hippocampal cells.

### Temporal synchronicity and behavioral-state specificity of dMEC-hippocampal replay

We then investigated the temporal synchronicity of hippocampal and dMEC replay coordination and assessed whether the coordination was observed during both rest as well as awake states.

Hippocampal-cortical replay coordination is thought to support the development of complementary cortical memory traces for long-term storage.^1^^,^^2^^,^^11^ If the dMEC-hippocampal replay coordination we observed here for theta-modulated dMEC cells indeed reflects this process, one may expect that the putative dMEC replay lags hippocampal replay—reflecting the projection of hippocampal memories to the cortex. To address this question, we shifted the spike times of dMEC cells by varying amounts (±80 ms) and computed the average coordination with hippocampal replay at each time shift. To note, to enable analysis of temporal synchrony at long time lags, this analysis was limited to long replay events (≥80 ms long). As expected, theta-modulated dMEC cells showed a clear peak in coordination if we assumed that the dMEC cells lagged the CA1 cells by 10 ms (Figure 3F). No such peak was observed for non-theta-modulated cells, whose coordination with hippocampal replay remained at chance irrespective of the time shift (Figure 3F).

**Figure 4:**
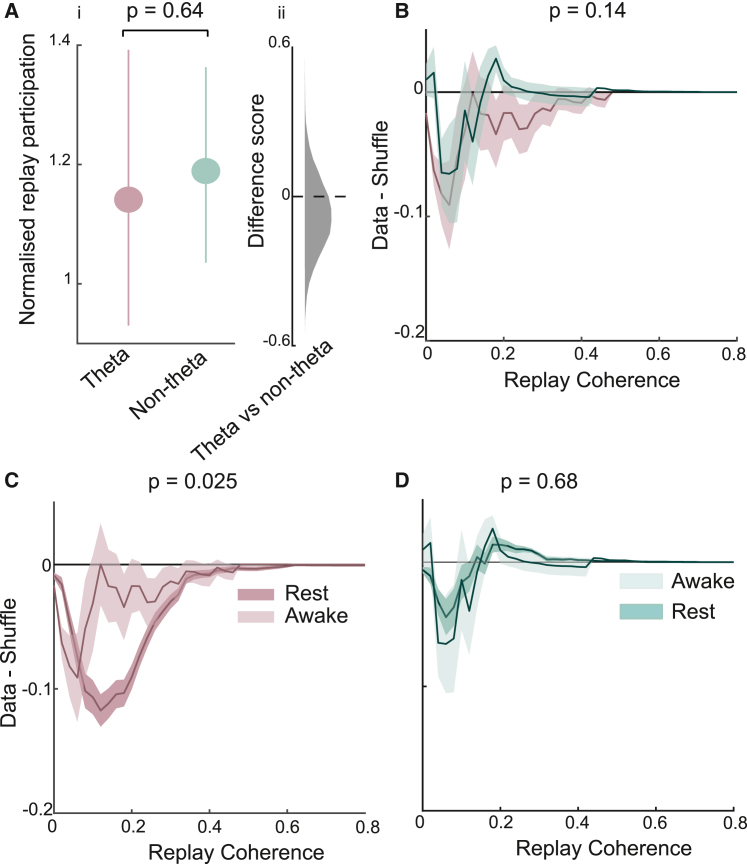
Behavioral-state specificity of dMEC-hippocampal replay (A) (i) Normalized participation of theta-modulated and non-theta-modulated dMEC cells in candidate awake hippocampal replay events. Error bars show the 95% CI. (ii) Histogram distribution of the bootstrapped difference scores. (B) Normalized awake replay coordination for theta-modulated and non-theta-modulated dMEC cells. Shaded area shows one SD. (C) Normalized replay coordination for theta-modulated dMEC cells during awake and rest periods. (D) Same as (C) but for non-theta-modulated cells. See also Figure S5.

Thus far, we have analyzed hippocampal replay coordination during rest periods. Indeed, offline replay is thought to play a privileged role in memory consolidation.^11^^,^^12^ However, replay is also known to occur during wakeful immobility periods.^32^^,^^33^ Importantly, this “online” replay has been purported to support different functions, such as planning and decision making,^13^^,^^15^^,^^16^ although this functional distinction is still debated.^34^ Thus, we sought to investigate whether dMEC cells are also coordinated with the hippocampal replay that occurs while the animals are immobile on the track (<1 cm/s movement speed). In contrast to the offline analysis, we found neither dMEC cell group displayed strong recruitment to candidate replay events (normalized replay participation: theta-modulated = 1.14 [SD = 1.37], non-theta-modulated = 1.19 [SD = 1.03]; theta versus non-theta-modulated cells, p = 0.64; Figure 4A). Indeed, only the dMEC cells that were not modulated by theta showed above-chance replay participation (non-theta-modulated cells, p = 0.0069; theta-modulated cells, p = 0.11). With regard to coordination with hippocampal replay trajectories, we also found that the two cell groups displayed similar levels of coordination (theta-modulated versus non-theta-modulated cell AUC, p = 0.14; Figure 4B). Although the coordination demonstrated by the theta-modulated sub-group did exceed chance levels (p = 0.017), it was significantly lower than that observed during offline replay periods (p = 0.025; Figure 4C). dMEC cells that did not exhibit theta-modulated activity showed no such behavioral-state-dependent change in their coordination with hippocampal replay (offline versus online replay coordination, p = 0.68; Figure 4D). To note, a similar behavioral-state-dependent change in replay coordination was observed using a different movement speed threshold (3 cm/s; Figure S5A). Thus, theta-modulated dMEC cells show accentuated coordination with hippocampal replay during offline periods—the behavioral period thought to be essential for the consolidation of new memories.

### dMEC-hippocampal replay synchrony requires and improves with learning

If hippocampal-dMEC replay coordination reflects learning, then one might expect the strength of hippocampal-dMEC replay synchrony to be modulated by the level of experience an animal has with a task. As such, we analyzed replay coordination separately for three different learning periods, namely, early (days 1 and 2), mid (days 3 and 4), and late (days 5 and 6). Importantly, performance on the task—measured by the number of incorrect turns at the corners of the maze—improved across these three periods (r = −0.37, p = 0.04). To note, this analysis only included the three animals that we recorded during the three learning periods so as to ensure a similar amount of data in each of the periods. However, the main results are replicated if all animals are included (Figure S5B).

**Figure 5:**
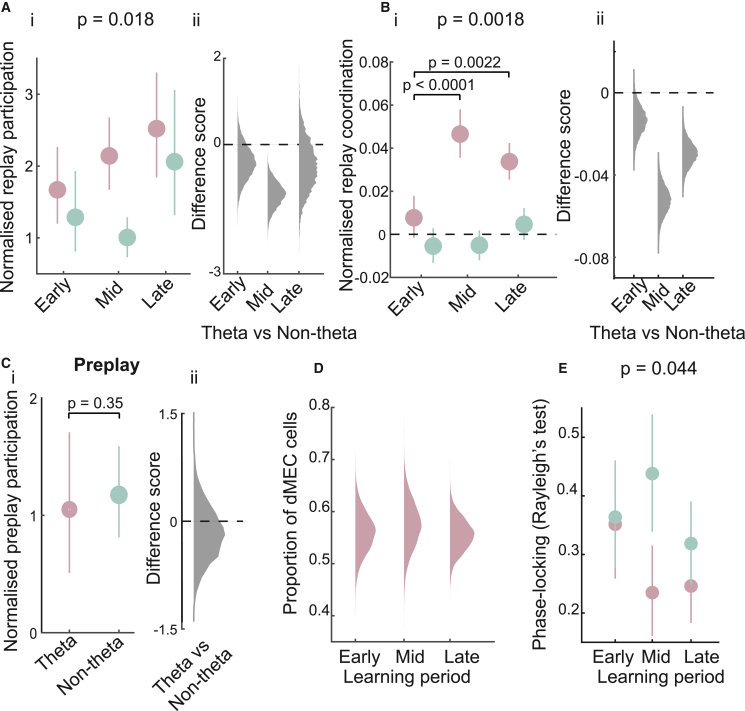
dMEC-hippocampal replay coordination of theta-modulated cells reflects learning (A) (i) Normalized participation of theta-modulated and non-theta-modulated dMEC cells in candidate replay events for different learning periods. Error bars show 95% CI. (ii) Frequency distribution of bootstrapped difference scores (theta-modulated − non-theta-modulated). (B) (i) Normalized replay coordination (data-shuffle) with 95% CI for the three learning periods. (ii) Frequency distribution for bootstrapped difference scores (theta-modulated − non-theta-modulated). (C) (i) Normalized participation of theta-modulated and non-theta-modulated dMEC cells in candidate hippocampal preplay events. Error bars show 95% CI. (ii) Frequency distribution of difference scores. (D) Bootstrapped frequency distribution of the proportion of dMEC cells classified as theta-modulated for the different learning periods. (E) Phase locking to hippocampal theta-band oscillations during different learning periods. Error bars show 95% CI. See also Figure S5.

In terms of participation in replay, we observed that theta-modulated dMEC cells were significantly more likely to participate in candidate replay events than expected by chance, for all learning periods (mean replay modulation: early = 1.67 [SD = 2.05], p = 0.0013; mid = 2.14 [SD = 1.8]; late = 2.52 [SD = 2.7], p < 0.0001; Figure 5A). Non-theta-modulated dMEC cells, however, did not display reliable participation in replay events across the different learning periods, with only the late learning period being associated with above-chance participation (mean modulation: early = 1.29 [SD = 1.85], p = 0.85; mid = 1.01 [SD = 0.82], p = 0.52; late = 2.06 [SD = 2.62], p = 0.0005; Figure 5A). Further, a linear mixed-effects analysis, where animal ID was included as a random effect, showed a significant experience-dependent increase in replay participation as the animals became more experienced with the task (*t*_(257)_ = 2.38, p = 0.018). In terms of coordination with replay trajectories, we found that theta-modulated dMEC cells showed marginally better coordination than expected by chance during the early learning period (p = 0.06, bootstrapped AUC) but robust coordination during the mid and late learning period (mid and late, p < 0.0001; Figure 5B). Non-theta-modulated dMEC cells, on the other hand, were not coordinated with replay during any learning period (early, p = 0.90; mid, p = 93; late, p = 0.11; Figure 4E). Again, a linear mixed-effects analysis confirmed the effect of experience on replay coordination (*t*_(1,369)_ = 3.13, p = 0.0018). Furthermore, the theta-modulated dMEC cells showed consistently higher coordination than their non-theta-modulated counterparts during all learning periods (early, p = 0.023; mid, p < 0.0001; late, p < 0.0001; Figure 5Bii). Finally, a post hoc pairwise comparison revealed that theta-modulated dMEC cells showed a reliable increase in replay coordination between the first and second, as well as the first and the third, learning period (1^st^ versus 2^nd^, p < 0.0001; 1^st^ versus 3^rd^, p = 0.0022; Bonferroni corrected), whereas non-theta-modulated cells showed no learning-related increase in replay coordination (all, p > 0.05).

The foregoing analysis shows that the preferential coordination of theta-modulated dMEC cells with hippocampal replay is already present in the early learning period. Thus, perhaps this preferential coordination reflects pre-existing connectivity between CA1 and theta-modulated dMEC cells. To address this question, we analyzed whether theta-modulated dMEC cells also showed preferential recruitment to hippocampal preplay events recorded during the rest session preceding the animals’ first encounter with the track. Neither group of dMEC cells showed a significant increase in their activity during candidate preplay events in the pre-sleep session (theta-modulated cells = 1.05 [SD = 1.54], p = 0.53; non-theta-modulated cells = 1.17, [SD = 1.05], p = 0.80; Figure 5C), and the degree of activity modulation did not differ between the two groups (p = 0.035). We could not analyze coordination with replay trajectories as we did not consistently find more preplay trajectories than expected by chance during this pre-sleep session. Thus, the preferential recruitment and synchronization of theta-modulated dMEC cells described above emerged as an animal was exposed to and acquired a novel spatial task and is thus likely to reflect experience-related plasticity in hippocampal-entorhinal circuits.

Finally, if the dMEC-hippocampal replay modulation for theta-modulated entorhinal cells reflects the coupling of these cells to the hippocampal theta rhythm, perhaps then the emergence of this coordination—and subsequent experience-dependent strengthening—reflects an experience-dependent increase in synchronization of dMEC cells to the hippocampal LFP. To address this question, we analyzed dMEC phase locking to the hippocampal theta rhythm during the three learning periods. Consistent with our hypothesis, we found the mean phase locking of theta-modulated dMEC cells to increase as the animals became more experienced with the task (mean phase locking: early = 0.35 [SD = 0.36], mid = 0.24 [SD = 0.27], late = 0.25 [SD = 0.29]; early versus mid, p = 0.023; early versus late, p = 0.033; Figure 5E), an effect not observed for non-theta-modulated dMEC cells (early = 0.37 [SD = 0.31], mid = 0.44 [SD = 0.31], late = 0.33 [SD = 0.31]; early versus mid, p = 0.87; early versus late, p = 0.31; Figure 5E). Further, a linear mixed-effects analysis confirmed the effect of learning period on phase locking (*t*_(330)_ = −2.02, p = 0.044). Indeed, phase locking to hippocampal theta by theta-modulated dMEC cells became as strong as the phase locking displayed by CA1 cells during the mid and late learning periods (theta-modulated dMEC versus CA1 phase locking: early, p = 0.013; mid, p = 0.65; late, p = 0.24). Importantly, this effect could not be explained by an increase in the number of theta-modulated dMEC cells, as the proportion of dMEC cells showing significant theta rhythmicity in their spike trains remained the same during the different learning periods (Figure 5D).

## Discussion

Influential theories posit that the formation of long-term memories relies on sub-second activity synchronization between hippocampal and cortical units during offline periods.^2^^,^^4^ Yet it has hitherto remained unclear how slow, behavioral-timescale activity patterns during encoding can lead to millisecond-level, cross-regional, offline synchronization. We show that this offline coordination may be mediated by dMEC cells locking to theta oscillations during encoding periods. Specifically, we found dMEC cells that showed spike-time entrainment in the theta band, coupled to hippocampal local field potential, were selectively recruited to hippocampal replay events. Further, our study shows for the first time that cross-regional synchronization is influenced by behavioral state—being accentuated during behavioral periods thought to be crucial for long-term learning (i.e., rest). Finally, we found the dMEC-hippocampal replay coordination of the task environment to be experience dependent, only emerging after an animal had physically explored a novel environment, and then displaying a learning-dependent increase that was mirrored by dMEC cells becoming increasingly entrained to the hippocampal theta band. To our knowledge, this study is the first to show that hippocampal-cortical replay coordination is directly related to learning. Furthermore, our study highlights a candidate mechanism—namely, shared oscillatory coupling during encoding—that may be responsible for this offline synchronization and, ultimately, the commissioning of memories to long-term storage.

The hippocampal theta rhythm has long been proposed to play a central role in extra-hippocampal communication^35^^,^^36^^,^^37^^,^^38^ as well as sequence-based plasticity.^36^^,^^39^ For example, phase locking of prefrontal cortical cells to the hippocampal theta band during awake periods is associated with their enhanced participation in awake SWRs,^40^ and theta-nested hippocampal cell sequences have been found to be required for faithful reactivations during post-task rest periods.^39^^,^^41^ However, this study is the first to show that theta may also play a pivotal role in the establishment of offline hippocampal-cortical replay synchronization—a process thought to lie at the heart of long-term memory formation. Although the precise mechanisms underlying theta sequence generation remain debated, they invariably build on precise spike-time synchronization of CA1 cells with ongoing theta-band oscillations, be it in the form of phase precession^20^ and/or phase locking.^18^^,^^21^^,^^22^^,^^24^ As such, we hypothesize that the dMEC theta phase locking we observed may reflect the emergence of distributed, hippocampal-dMEC theta sequences.

Our study also showed that hippocampal-dMEC replay coordination was specific to offline periods. These results suggest that different replay circuits may operate during distinct behavioral states, as suggested by previous authors.^42^^,^^43^^,^^44^ The benefit of having such diverse circuits may be that it allows replay to serve multiple functions. During awake periods, replay may support ongoing behavior (e.g., by supporting planning), leading to distinct routes of information propagation compared with rest periods, when replay may primarily serve to strengthen newly formed memory traces. In the latter case, information may be preferentially routed via the dMEC, given its extensive projections to the cortex,^45^ allowing complementary cortical memory traces to be formed over time.

Finally, to study the role of hippocampal-dMEC replay coordination in learning, we trained animals to run for food reward on a Z-shaped track. Although the task may not depend on the hippocampus, we believe insights gained by investigating the relationship between replay coordination and performance on simple spatial tasks may still deepen our understanding of the neuronal mechanisms supporting hippocampal-dependent learning (such as episodic and spatial memory). Namely, as replay sequences as well as hippocampal-cortical interactions have been consistently observed during rest periods following behavioral epochs on a variety of tasks—ranging from linear tracks^32^^,^^33^^,^^46^ to spatial memory tasks^8^^,^^13^—it is likely that insights gained from learning on these simple tasks, such as the one we have used, can be transferred to more complex episodic/spatial memory tasks. Consequently, our findings may represent a model of how long-term explicit memory formation may emerge.

In conclusion, this study highlights and extends the role of the theta rhythm in information propagation and plasticity in hippocampal-cortical circuits, particularly in the domain of sequence plasticity. Further, finding a sub-population of cells residing in the deep layers of the MEC that preferentially participate in hippocampal replay is consistent with the anatomical position of this subregion—i.e., it is the principle output center of the hippocampus^6^^,^^47^—and extends emerging theories pointing to the dMEC as a critical subregion supporting the effective transfer of memories to the cortex.^45^

## STAR★Methods

### Key resources table

**Table:** 

REAGENT or RESOURCE	SOURCE	IDENTIFIER
**Deposited data**		

Dataset used in the present study	Previously published and described^5^	https://zenodo.org/record/5566548

**Experimental models: Cell lines**		

Lister hooded rats	Charles River	RRID: RGD_2312466; https://www.criver.com/products-services/basic-research/find-amodel/lister-hooded

**Software and algorithms**		

MATLAB	Mathworks, MA	RRID: SCR_001622; https://uk.mathworks.com/products/matlab.html
Tint spike sorting software	Axona	Product code: COMP/TINT01, http://axona.com/products

**Other**		

Recording system (pre-amp and systems unit)	Axona	Product code: Dacq/USB64, http://axona.com/products
Omnetic connectors (microdrive assembly)	Genalog	Product code: A79026-001, https://genalog.com/genaloglinecard/omnetics/
Single-screw mouse Microdrive	Axona	Product code: MDMR-01M1, http://axona.com/products
4 x 16 channel headstage preamplifiers	Axona	Product code: HS-116M1D, http://axona.com/products
4 x fine wire tethers	Axona	Product code: HS16-CAB3, http://axona.com/products
Microwire (17um, platinum iridium)	California Fine Wire Company	Product code: 100167, https://www.calfinewire.com/datasheets/100167-platinum10iridium.html
NanoZ plating equipment	Multichannel System	nanoZ, https://www.multichannelsystems.com/products/nanoz

### Resource availability

#### Lead Contact

Further information and requests for resources should be directed to and will be fulfilled by the Lead Contact, H. Freyja Ólafsdóttir (freyja.olafsdottir@donders.ru.nl).

#### Materials availability

This study did not generate new unique reagents.

### Experimental model and study participant details

Six male Lister Hooded rats were used in this study. All procedures were approved by the UK Home Office, subject to the restrictions and provisions contained in the Animals (Scientific Procedures) Act of 1986. All rats (330-400g at implantation) received two microdrives, each carrying eight tetrodes of twisted 17μm HM-L coated platinum iridium wire (90% and 10%, respectively; California Fine Wire), targeted to the right CA1 (ML: 2.2mm, AP: 3.8mm posterior to Bregma) and left medial entorhinal cortex (MEC) (ML = 4.5mm, AP = 0.3-0.7 anterior to the transverse sinus, angled between 8-10°). Wires were platinum plated to reduce impedance to 200-300kΩ at 1 kHz. After rats had recovered from surgery they were maintained at 90% of free-feeding weight with ad libitum access to water, and were housed individually on a 12-hr light/dark cycle, with experiments conducted during the light period.

### Method details

#### Recording

Screening was performed post-surgically after a 1-week recovery period. An Axona recording system (Axona) was used to acquire the single-units and positional data (for details of the recording system and basic recording protocol see Ólafsdóttir et al.^5^). The position and head direction of the animals was inferred using an overhead video camera to record the location of two light-emitting diode (LED) mounted on the animals’ head-stages (50Hz). Tetrodes were gradually advanced in 62.5um steps across days until place cells (CA1) or grid cells (MEC) were found.

#### Experimental apparatus and protocol

The experiment was run during the animals’ light period to encourage quiet restfulness during the rest session. Animals ran on a Z-shaped track, elevated 75cm off the ground with 10cm wide runways. The two parallel tracks of the Z (190cm each) were connected by a diagonal section (220cm). The entire track was surrounded by plain black curtains with no distal cues. During each track session, animals were required to complete laps on the elevated Z-track, traversing each of the three tracks in order before returning in the other direction. At each end and corner, animals received a sweetened rice grain. Importantly, reward was withheld if the animal made an incorrect turn at the corners. Four animals (R2142, R2192, R2198, and R2217) were trained to run on the track for 3 days before recording commenced. For the other animals (R2242, R2335, R2336, R2337), recordings were made from the first day of exposure to the Z-track task.

Following the track session, rats were placed in the rest enclosure for 90 minutes. The rest enclosure consisted of a cylindrically shaped environment (18cm diameter, 61cm high) with a towel placed at the bottom and was located outside of the curtains which surrounded the Z-track. Animals were not able to see the surrounding room while in the rest enclosure. Prior to the experiment, rats had been familiarized with the rest enclosure for at least 7 days. Animals R2242, R2335, R2336 and R2337, were also placed in the rest enclosure for 90 minutes prior to the first Z-track session on day 1 of the experiment. Recordings from this ‘pre-rest’ session were not analyzed as part of this study. Following the rest session, animals completed a 20min foraging session in an open field environment. This session was included to enable functional classification of MEC cells and was not analyzed in the current study.

#### Data inclusion/exclusion

Sessions recorded on days1-6 were submitted for analysis. One session was excluded as result of data loss caused by the headstages becoming disconnected from the microdrives during the rest session (R2336 day4) and one due to absence of an eeg recording (R2142, day4). In total 22 sessions were submitted for further analysis.

### Quantification and statistical analysis

#### Theta modulation analysis

Theta modulation was computed for each dMEC cell individually. Specifically, each cell’s spiking activity (1ms bins) density was computed, autocorrelated (-30 to 30 sec) and convolved (20 ms). Next, theta-band modulation score was calculated by comparing the strength of theta-band (5-12Hz) against the strength of broadband (20-125Hz) and then Hilbert-transformed to obtain their respective amplitude envelope. Theta- and broad-band scores were then obtained by the theta and broad-band mean amplitude ratio within the -1 to 1 second window. The ratio between amplitude in theta and broad-band was used to theta-modulated cells. To compute statistical significance we randomly permuted the spike times of individual cells within the sessions time window. dMEC cells whose theta modulation score was above or equal the 97.5^th^ percentile of the permutation distribution were considered to be theta-modulated.

To note, only cells whose mean firing rate did not exceed 7Hz were included and whose activity fell into one of the following functional cell type categories: grid cells, head direction cells, border cells, conjunctive cells, other spatial cells. The method for each classification is described below.

#### Theta phase locking

dMEC cells were scored by their locking to ongoing hippocampal theta. To do so, we first identified the electrode in the CA1 region with strongest theta (5-12Hz) to delta (2-4 Hz) ratio. We filtered the selected CA1 channel’s signal in the theta band (finite impulse response filter, ‘Hamming’ window), Hilbert-transformed it and extracted its instantaneous phase, allowing to identify the theta phase of each spike.

Only spikes elicited when the animal’s running speed was above 3cm/s were included in this analysis. Theta coherence was computed via the Rayleigh test of uniformity on each cell’s spiking theta phases, where a lower value indicates better locking. Statistical significance between cell groups was computed by bootstrapping Rayleigh test scores and computing difference scores between the bootstrapped data.

#### Hippocampal replay

Ratemaps for the Z-track were generated after first excluding areas in which the animals regularly performed non-perambulatory behaviors (e.g. eating, grooming); the final 10cm at either end of the track and 5cm around each of the two corners. Similarly, periods when the animals’ running speed was <3cm/s were also excluded. To generate ratemaps, the animals’ paths were linearized, dwell time and spikes binned into 2cm bins and smoothed with a Gaussian kernel (σ = 5bins), firing rates were calculated for each bin by dividing spike number by dwell time. Separate ratemaps were generated for runs in the outbound and inbound directions. To identify place fields, spatial bins whose rate exceeded the mean firing rate of the cell on the track were only considered. Hippocampal cells were classified as place cells if they exhibited firing greater than its mean rate for 20contiguous bins and if the peak firing rate was >1hz. These cells were submitted to further analysis. Interneurons, identified by narrow waveforms and high firing rates, were excluded from all analyses

Putative replay events were identified based on the activity of hippocampal cells using a similar method to Ólafsdóttir et al.^5^ To identify replay events, multi-unit (MU) activity from CA1 cells were binned into 1ms temporal bins and smoothed with a Guassian kernel (σ = 20ms). Time points when the MU activity exceeded 3 standard deviations above the mean MU activity in the rest session were flagged as candidate replay events. The start and end points of each candidate event were determined as the time when the MU activity fell back to the mean. Events less than 40ms long and which contained less than 15% of the recorded CA1 population (or 5 cells, which ever was greater) were rejected. Further, for the awake replay analyses we excluded all events that occurring when the animals’ average movement speed exceeded 1cm/sec. We also used a 3cm/sec movement threshold to ensure the results were not sensitive to the exact value of this threshold.

For position decoding of candidate events a Bayesian framework^5^ was used to calculate the probability of the animal’s position in each spatial bin given the observed spikes; the posterior probability matrix. Note, two posterior probability matrices were generated for each event, one for inbound runs and one for outbound runs. Spike data was divided up into 10ms temporal bins, and decoding was carried out on each bin separately.

To score the extent to which putative trajectory events represented a constant speed trajectory along the linearized Z-track we applied a line-fitting algorithm.^5^ Lines were defined with a gradient (V) and intercept (c), equivalent to the velocity and starting location of the trajectory. The goodness of fit of a given line was defined as the proportion of the probability distribution that lay within 30cm of it. Specifically where P is the probability matrix:

(Equation 1) $$R\left(V,c\right)=\frac{1}{n}\sum_{\tau =0}^{n-1}P\left(\left|x\left(t\right)-\left(V.t.T+c\right)\right|\leq d\right)$$

where t indexes the time bins of width and d is set to 30cm. was maximized using an exhaustive search to test all combinations of V between -50m/s and 50m/s in 0.5m/s increments (excluding slow trajectories with speeds > -2m/s and < 2m/s) and c between -15m and 21m in 0.01m increments.

To assess candidate replay events for significance we carried out a spatial field shuffle of the place cell ratemaps. Specifically, each ratemap was ‘rotated’ by shifting it relative to the track by a random number of bins drawn from a flat distribution between 1 and the length of the track minus 1 bin. The ratemap for each cell was rotated independently and in each case trailing bins were wrapped around to ensure an equal number of bins were used for each shuffle. This process was repeated 100 times for each event and for each shuffle we recalculated a goodness of fit measure (as described above). This enabled us to estimate the probability of obtaining a given event by chance. Replay trajectory events were defined as those with an individual p-value below 0.025 – to control for multiple comparisons for in- and outbound runs.

#### dMEC-hippocampal replay coordination

To analyze dMEC coordination with replay events we carried out two kinds of analyses. First, the proportion of candidate replay events (defined as those events submitted to trajectory decoding) a dMEC cell was active in was estimated for theta-modulated and non-modulated cells. To control for possible differences in baseline activity levels between the two cell types we computed the chance participation score for each individual cell by shuffling the event times of candidate events. The mean of the shuffle was then divided by the participation score in the real data. On this measure a value above 1 indicates the cell is active in more events than expected by chance. To estimate if individual cells were significantly modulated by candidate replay events the 95% confidence interval for each cell’s shuffle distribution was estimated. To assess if theta-modulated and non-modulated cell groups showed significant modulation the participation scores were bootstrapped (N=10,000). The difference between the two groups (theta – non-theta) was then assessed for statistical significance by computing the difference between the bootstrapped distributions and to estimate the p-value we counted the number of differences scores that fell below 0 (indicating non-theta participation scores were higher than theta participation scores) and divided this number by the total number of bootstraps. To note, we also carried out alternative activity modulation analyses where we assessed if the two dMEC groups differed in how many spikes they emitted on average during candidate replay events and whether the two groups differed in terms of the proportion of neurons that displayed significant modulation on each measure. Statistical testing was done as before.

To investigate replay coordination between dMEC and CA1 cells we applied the same framework as we did in previous work.^5^ Namely, a Bayesian decoding was done on dMEC cell spikes (to note, this analysis was done separately for theta-modulated and non-modulated dMEC cells). Hence, for each replay event we also calculated a posterior probability matrix based solely on the observed dMEC cell spikes. Rather than fitting straight-line trajectories to the dMEC cell posteriors, we compared the best-fit line (i.e. hippocampal replay trajectory) from the concurrently recorded CA1 posterior. Specifically, the dMEC-CA1 cell replay coherence score was calculated using the slope and intercept parameters of the best-fit line of the accompanying CA1 event. This value we used to index replay coordination between hippocampal and dMEC cells. To estimate statistical significance of the observed coherence scores we used a cell ID shuffle. Namely, a shuffle distribution was generated by randomly permuting the cell IDs of dMEC cells so that cells were allocated a random ratemap (from other dMEC spatial cells recorded in the session). The line fitting procedure to estimate dMEC-place cell replay coherence, described above, was re-run. To assess the statistical significance of the obtained distribution of coherence scores against the shuffle we bootstrapped the data distribution 10,000 times, computing the cumulative distribution and the corresponding area-under-the-curve (AUC, i.e. the sum of the cumulative distribution) for each bootstrap. Difference scores between each of the 10,000 AUC scores obtained from the bootstrapped data and the shuffle distribution were computed and the 95% confidence interval estimated based on these difference scores. To obtain a p-value we counted the number of difference scores (data-shuffle) that fell below zeros (indicating better coherence in the shuffle relative to the data) and divided this number by the number of bootstraps (10,000). To compare replay coordination between theta-modulated and non-modulated dMEC cells we computed difference scores between bootstrapped AUC scores for the two cell types. To note, normalized AUC scores (data-shuffle) where used to control for dMEC sub-group specific differences.

Second, we applied a spatial field shuffling procedure. This procedure was similar to the shuffling procedure used for hippocampal events. Specifically, each dMEC cell ratemap was shuffled by shifting it relative to the track by a random number between 10 and the length of the track minus 10 bins. The ratemap for each cell was rotated independently and trailing bins were wrapped around to ensure an equal number of bins were used for each shuffle. This process was repeated 100 times for each event. For each shuffle, the dMEC-hippocampal replay coherence score was calculated using the slope and intercept parameters of the best-fit line of the accompanying hippocampal event (unshuffled). To assess statistical significance we used an AUC test as described above.

Third, we applied a spike time shuffle, following recommendation by Trimper et al.^30^ and also in line with our previous work.^5^ Specifically, the spike time of each dMEC cell was randomly permuted by shifting the timing of spikes from individual cells by a random number between 5ms and the length of the events minus 5ms. Shuffled spike times that exceeded the event were than wrapped around the start of the event. This process was repeated a 100 times for each event. Statistical significance was estimated following the procedure described above.

We also repeated the analysis using a more stringent threshold for theta modulation. Namely, rather than counting dMEC cells whose theta vs broadband ratio exceeded the 97.5^th^ percentile of its own shuffle distribution as theta-modulated cells we used the 99^th^ percentile instead. Further, we assessed if replay coherence varied linearly with the degree of theta modulation displayed by dMEC cells. To this end, we divided the entire (theta and non-theta) dMEC cell population into quartiles based on their theta modulation score. For each quartile we then carried out the same replay coherence (and modulation) analysis as described above.

Further, we repeated the analyses after excluding dMEC cells with large spatial firing fields. For this, we used two different thresholds: 100cm and 150cm. In each case, all dMEC cells whose every spatial firing field exceeded these thresholds were excluded.

To ensure differences in replay coordination between theta-modulated and non-modulated cells could not simply be explained by differences in the number of cells belonging to each category we carried out a down-sampling analysis. Specifically, we down-sampled the theta-modulated cell population to match that of the non-modulated cell groups by removing at random cells from the theta-modulated group. For each down-sampling iteration, we obtained a bootstrapped distribution of data-shuffle AUC scores. This process we repeated a 100 times and then the average of the down-sampled bootstrapped scores was obtained. These bootstrapped difference scores were then compared to the difference scores we obtained for the non-theta-modulated cell group.

To analyze replay coherence for forward and reverse replay events separately we identified replay events with a positive slope; these were classified as forward replay.

To assess the temporal synchronicity of dMEC and hippocampal replay trajectories we shifted the dMEC spike times by varying amount ranging from -80ms to +80ms. We carried out these time shifts in 10ms time bins and for each time shift we computed the average replay coordination.

#### Experience-dependent analysis

To analyze change in dMEC-CA1 replay coordination as a function of experience with the task, the data was divided into three learning periods: early (days1-2), mid(days3-4) and late(days5-6). For each learning period the mean dMEC-CA1 replay coordination or activity modulation (replay participation) was calculated and subtracted from the mean obtained from the shuffle distribution for that learning period (data-shuffle). To estimate statistical significance the normalized replay coherence/participation scores were bootstrapped (N=10,000) and the mean computed for each iteration of the bootstrap. The difference between the bootstrapped data and the mean of the shuffle was then computed to assess for statistical significance, using the same procedure for computing p-values as described above. A similar procedure was used to assess changed in dMEC locking to hippocampal theta. A Bonferroni correction was applied to control for multiple comparisons between different learning periods.

To note, for this analysis only data from three animals (R2335, R2336, R2337) were used as these participated in each of the learning periods. However, the main results remained the same if we included all animals.

#### Functional classification of dMEC cells

dMEC cells were classified as grid, head direction, border or other spatial cells using standard metrics, described below. Functional classification was done based on activity recorded during open field sessions that occurred after the post-sleep session. We repeated the cell type classification (bar classification of grid cells) using data recorded on the track to make sure our results were not sensitive to which data was used for classification.

dMEC cells were classified as grid cells using a shuffling procedure similar to that applied elsewhere. Specifically, the hexagonal regularity of each cell was assessed using the ‘standard’ gridness measure (Hafting, 2006). The values calculated for each cell were compared with a null distribution of 100 values obtained by calculating the gridness values of data in which the cell’s spike train had been randomly permuted relative to the position of the animal by at least 30s. A cell was considered to be a grid cell and admitted to the main analysis if its standard or modified gridness value exceeded the 95th percentile of the matching null distribution.

Direction modulation was assessed by calculating the Kullback-Leibler (KL) divergence between the cell’s polar rate map and a uniform circular distribution with equal mean:

(Equation 2) $$D_{KL}=\sum_{i}\frac{\tau_{1}\left(i\right)\log\left(\tau_{1}\left(i\right)\right)}{\tau_{2}\left(i\right)}$$

Where τ1(i) is the value in the ith bin of a polar rate map normalized to have area 1 (as a probability distribution) and τ2(i) is the ith bin of a uniform probability distribution with the same number of bins as τ1. Grid cells with KL divergence greater than 0.10 were considered to be directional.

Border score was computed as previously described.^48^ In summary, each cell’s firing fields were estimated by identifying groups of continuous spatial bins (bin size = 2cm) where the firing rate was above 30% of the cell’s peak firing rate and smaller than 70% of the arena’s area. Next, a border score (in the -1 to 1 range) was computed for each boundary individually by computing the relation between the firing field’s extent and mean distance to the wall. As in Solstad et al. (2008), cells with a border score above 0.5 were considered border cells.

Spatial modulation was assessed using Skagg’s information.^49^ Cells whose Skaggs information (bits/spike) exceed 1 were considered as spatial cells. To note spatial cells were those cells that were not classified as any of the other spatial cell types described above.

To compare differences in representation of the distinct functional cell types for the theta-modulated and non-modulated cell groups we bootstrapped the proportions of each cell type for the two groups and computed difference scores and computed p-values as described previously.

#### dMEC-CA1 field overlap

In order to account for potential confounds derived from similar spatial tuning between dMEC and CA1 cells, we quantified the overlap of each cell pair rate maps during Z-track for each running direction (in- and out-bound). Only spatial bins with positive rates were included. dMEC-CA1 field overlap was scored by computing the correlation coefficient (Pearson-r test) between the two cells rate maps. To assess if theta-modulated dMEC cells displayed greater spatial firing field overlap with CA1 cells than non-theta-modulated cells we bootstrapped the ratemap correlation distributions for each group and then computed difference scores.

#### Performance analysis

To estimate performance on the Z-track, we counted the number of incorrect turns on the corners of the track and divided this by the total number of laps completed by an animal in a session. This measure gives the proportion of incorrect corner turns – a lower number indicate better performance. To assess if performance improved with experience we correlated proportion of incorrect turns with the number of days of experience using a Pearson correlation. Given we assumed an improvement with experience we report the statistics of a one-sided test.

#### Linear mixed effects analysis

To control for animal related differences in our main results we applied a linear mixed effects model. Specifically we used the fitlme function in Matlab where animal ID was included as a random effect (intercept).

#### Histology

Rats were anesthetized (4% isoflurane and 4L/min O2), injected intra-peritoneal with an overdose of Euthatal (sodium pentobarbital) after which they were transcardially perfused with saline followed by a 4% paraformaldehyde solution (PFA). Brains were carefully removed and stored in PFA which was exchanged for a 4% PFA solution in PBS (phosphate buffered saline) with 20% sucrose 2-3 days prior to sectioning. Subsequently, 40-50μm frozen coronal sections were cut using a cryostat, mounted on gelatine-coated glass slides and stained with cresyl violet. Images of the sections were acquired using an Olympus microscope, Xli digital camera (XL Imaging). Sections in which clear tracks from tetrode bundles could be seen were used to confirm CA1 recording locations.

## Data Availability

• All data analyzed in this study are publicly available from Zenodo (https://zenodo.org/record/5566548) • This paper does not report original code • Any additional information required to reanalyze the data reported in this paper is available from the lead contact upon request

## Supplemental information

Document S1. Figures S1–S5

Document S2. Article plus supplemental information
