# Supplementary material for: Theta-band phase locking during encoding leads to coordinated entorhinal-hippocampal replay
Source: Curr Biol. 2023 Nov 6;33(21):4570–4581.e5. doi: 10.1016/j.cub.2023.09.011 (PMC10629661; doi:10.1016/j.cub.2023.09.011)
Supplement: Document S1. Figures S1–S5 [file mmc1.pdf]

Current Biology, Volume *33*

Supplemental Information

Theta-band phase locking during encoding leads to coordinated entorhinal-hippocampal replay

Diogo Santos-Pata, Caswell Barry, and H. Freyja Ólafsdóttir

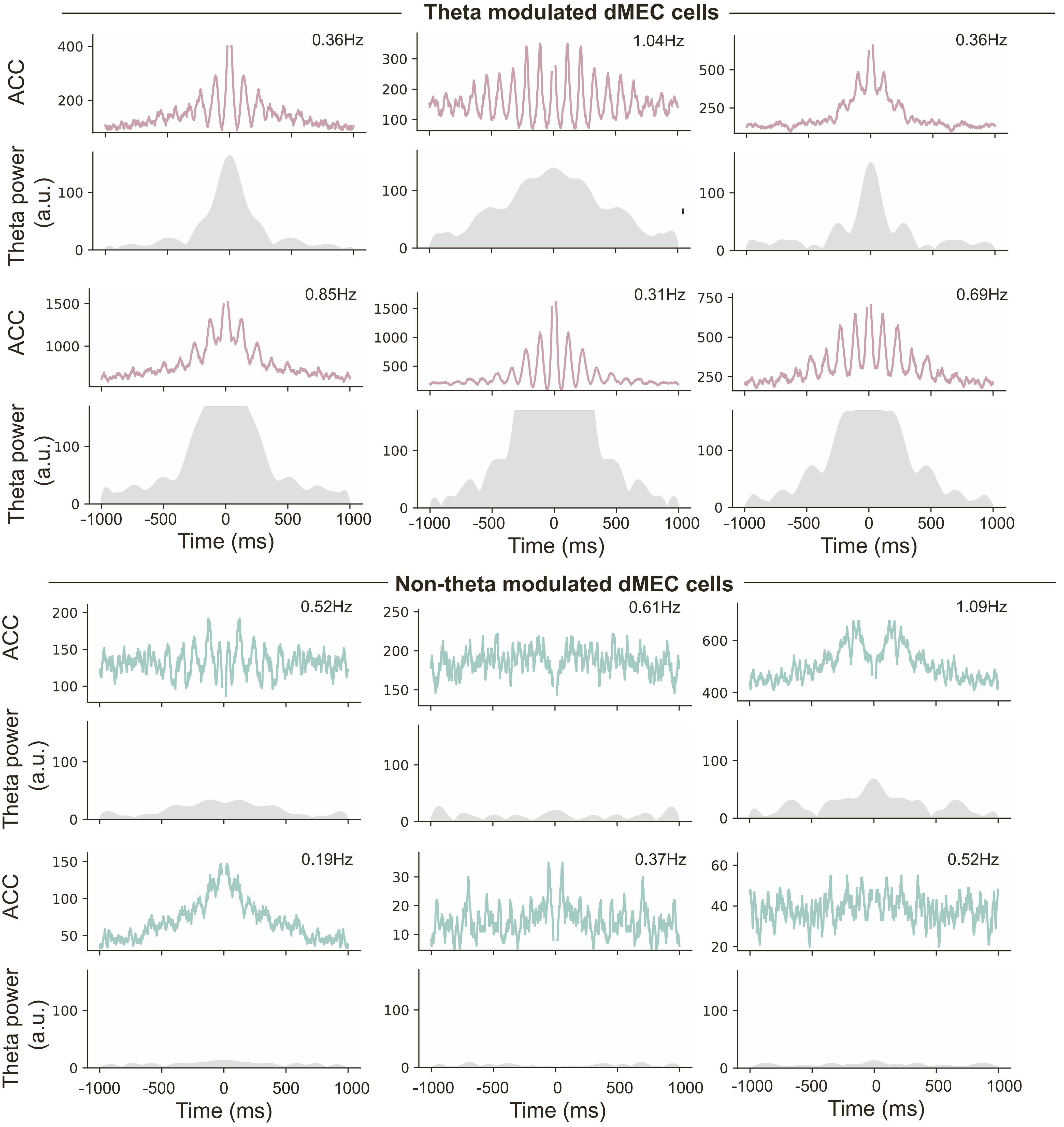

Figure S1. Theta-band rhythmicity in dMEC cells. Related to Figure 1. Top panels: autocorrelograms for theta-modulated (pink) and non-modulated (green) dMEC cells, top right shows average firing rate (Hz). Bottom panel: power in the theta-band (5-12Hz) in the autocorrelogram.

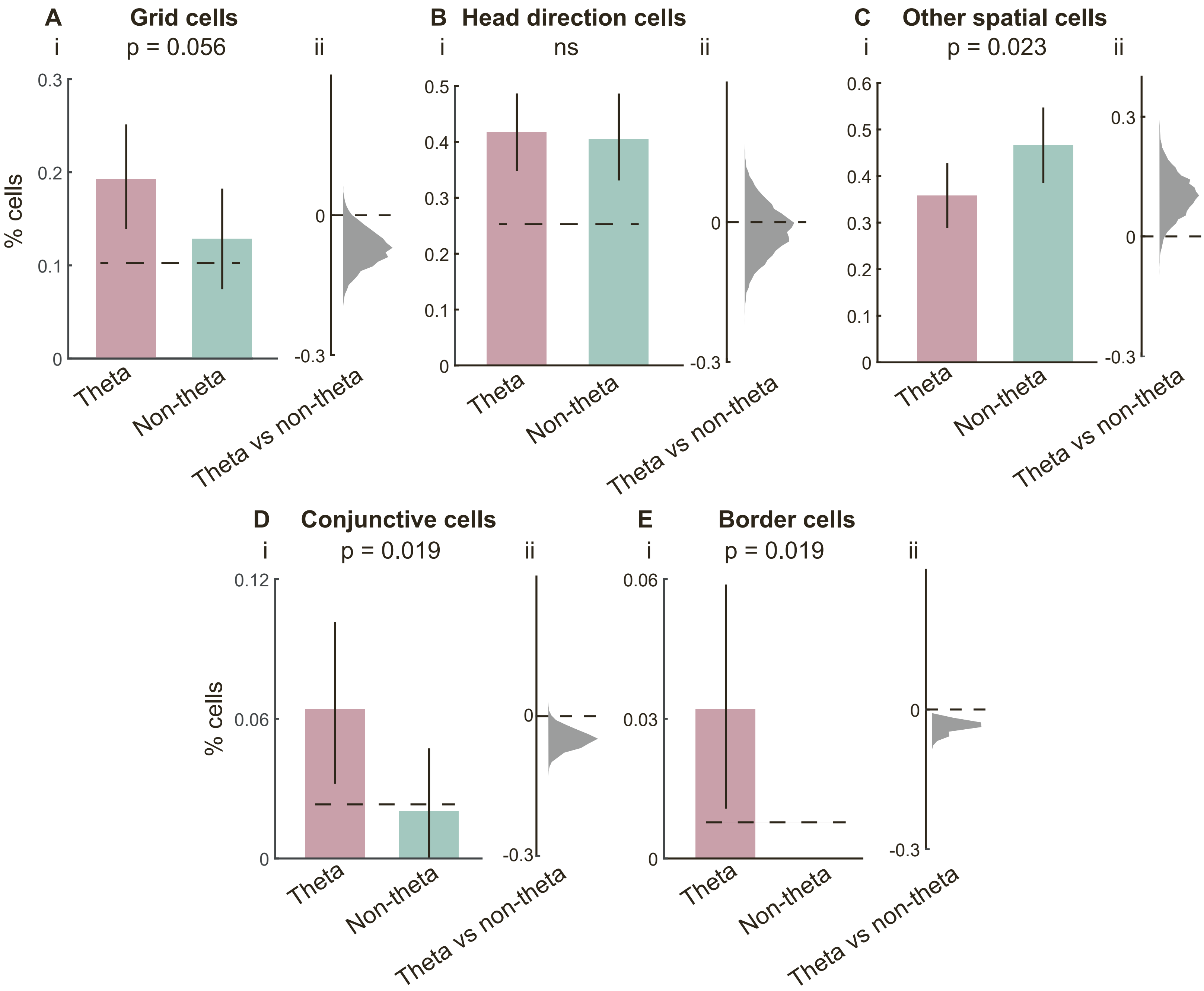

Figure S2. Representation of distinct functional cell types in the dMEC. Related to Figure 1. (A) (i) Proportion of dMEC theta-modulated (pink) and non-modulated (green) dMEC cells that qualify as grid cells. (ii) Histogram distribution of bootstrapped difference scores. (B-E) Same as (A) but for head direction, other spatial, conjunctive and border cells.

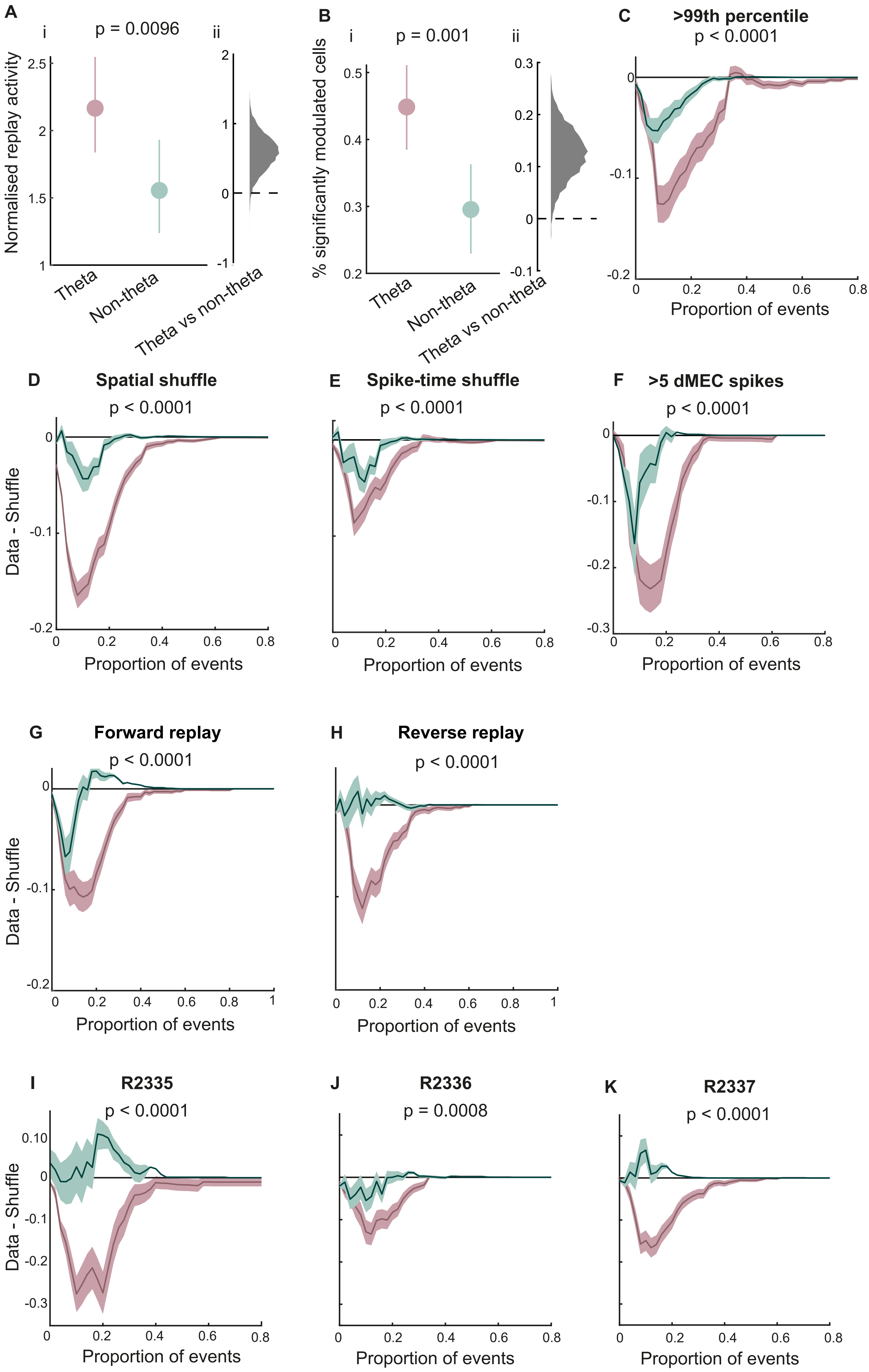

Figure S3. Hippocampal-dMEC replay coordination: Alternative and control analysis. Related to Figure 2. (A) (i) Mean normalised activity modulation of dMEC theta modulated (pink) and non-modulated (green) cells. Error bars show 95% CI. (ii) Kernel density of bootstrapped difference scores. (B) (i) Proportion of dMEC theta modulated and non-modulated cells that are significantly modulated by replay events, using measure from (A). Error bars show 95% CI. (ii) Bootstrapped difference scores. (C) Normalised (data-shuffle) replay coordination between hippocampal and theta and non-theta modulated dMEC cells using a more stringent threshold for theta modulation (99th percentile). Shaded area shows 1SD of bootstrapped data. (D-K) Same as (C) but using an alternative spatial field (D) and spike time shuffle (E), only including replay events with at least 5 dMEC spikes (F), limiting the analysis to forward (G) or reverse (H) replays, and carrying out the analysis for individual animals that had at least 10 dMEC cells in the two sub-groups (I-K)

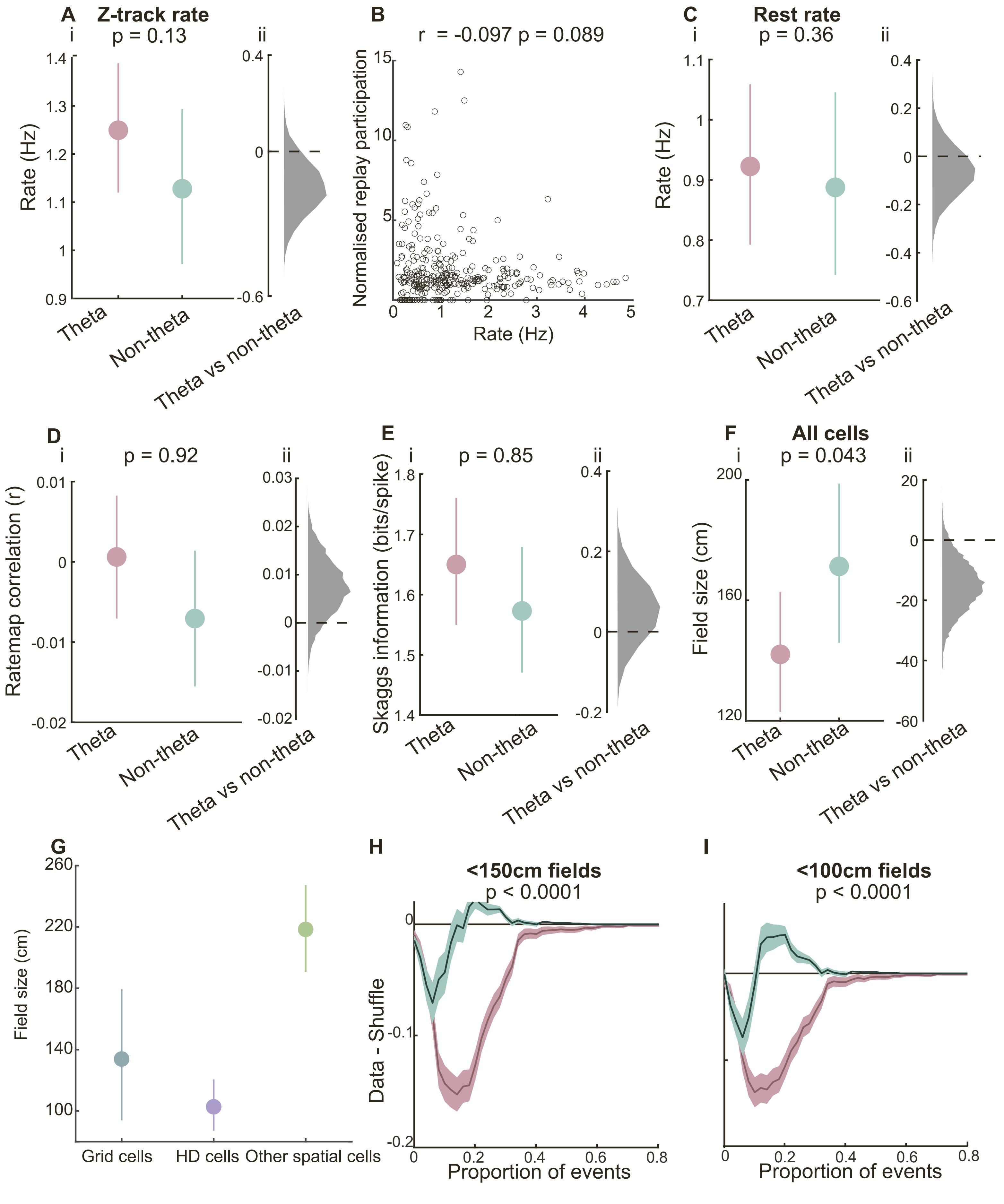

Figure S4. Preferential coordination of theta modulated dMEC cells with hippocampal replay trajectories is not confounded by functional and activity differences between theta-modulated and non-modulated dMEC cells. Related to Figure 3. (A) (i) Mean firing rate of theta modulated (pink) and non-theta modulated dMEC cells on the Z-track. Error bars show 95% CI. (ii) Histogram of bootstrapped difference scores. (B) Scatter plot between average firing rate (on the track, x-axis) and replay participation score (y-axis). (C) Same as A but for activity recorded during rest periods. (D-F)Same as (A) and (C) but showing average ratemap correlations between dMEC and CA1 cells (D),Skaggs information (E) and field size (F). (G) Average field size for grid, head direction and other spatial cells. (H) Normalised(data-shuffle) dMEC-hippocampal replay coordination after removing dMEC cells with large (>=150cm) spatial firing fields. Shaded area shows 1SD of bootstrappe data. (I) Same as H but using an alternative field size threshold (100cm).

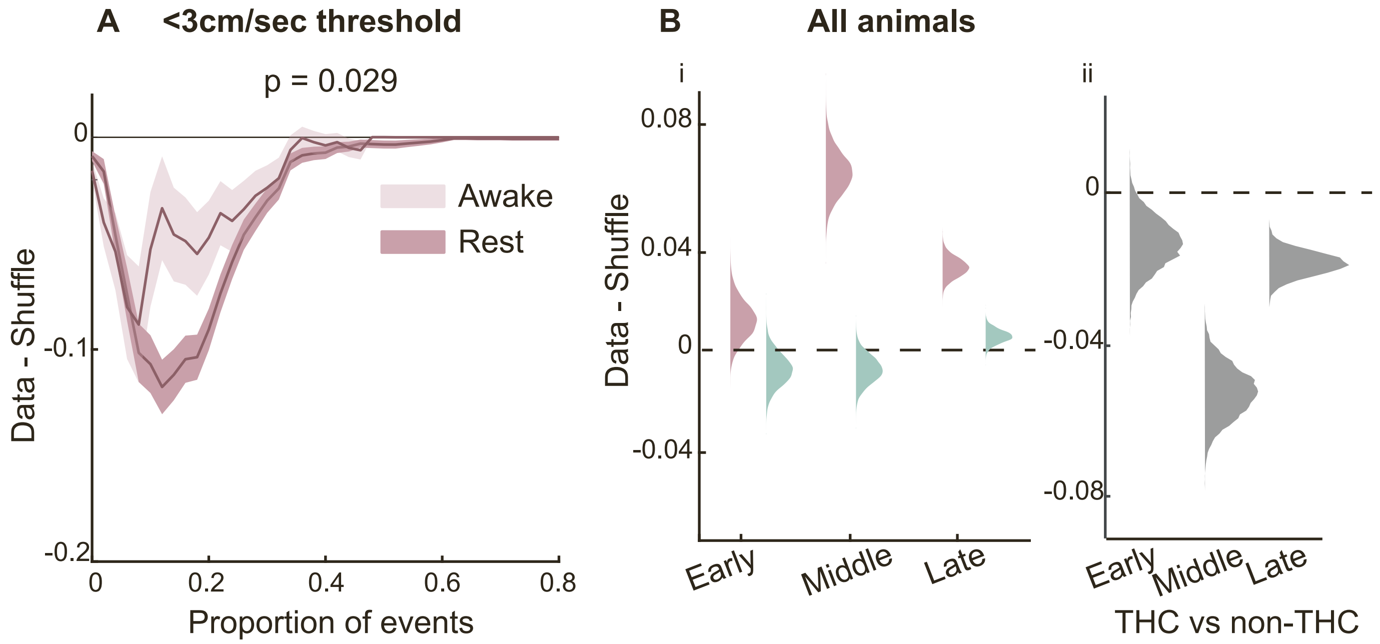

Figure S5. Alternative analyses for behavioural-state specigivity and experience-dependent increase in dMEC-hippocampal replay coordination. Related to Figures 4 and 5. (A) Normalised replay coordination for theta modulated dMEC cells during awake (light pink) and rest (dark pink) periods using an alternative movement threshold (<3cm/sec). Shaded are shows 1SD of bootstrapped data. (B) (i) Normalised (data-shuffle) replay coordination for theta-modulated (pink) and non-modulated (green) dMEC cells during distinct learning periods. Note, analysis includes all animals. (ii) Frequency distribution of bootstrapped difference scores between theta and non-theta modulated dMEC cells for individual learning periods.
